# Contraceptive Awareness and Its Usage Practices Among Adults Using Online Dating Apps in Chennai, Tamil Nadu: A Cross-Sectional Study

**DOI:** 10.7759/cureus.85166

**Published:** 2025-06-01

**Authors:** Jeffrey Joseph, Subhashini Viswanath, Gokul G

**Affiliations:** 1 Community Medicine, Sree Balaji Medical College and Hospital, Bharath Institute of Higher Education and Research, Chennai, IND; 2 Community Medicine, Meenakshi Medical College Hospital and Research Institute (MMCHRI), Meenakshi Academy of Higher Education and Research (MAHER), Kanchipuram, IND

**Keywords:** contraceptive agents, mobile applications, sexual behavior, sexual partners, unsafe sex

## Abstract

Introduction

Online dating apps have become pivotal for adults to find relationships, explore their sexual desires, and overcome loneliness. These users sometimes indulge in reckless and unsafe sexual practices, which may lead to unplanned pregnancies and sexually transmitted infections (STIs), thus emphasizing the importance of contraceptive use.

Methodology

This community-based cross-sectional study was conducted among 270 study participants in Chennai, Tamil Nadu. Study participants were selected by the snowball sampling technique. A pretested, semi-structured questionnaire was used to collect data and was analyzed using MS Excel (Microsoft Corporation, Redmond, Washington, United States) and IBM SPSS Statistics for Windows, Version 21 (Released 2012; IBM Corp., Armonk, New York, United States).

Results

The prevalence of contraceptive usage among online dating app users was found to be 67%. Factors like upper/upper-middle socioeconomic class (adjusted odds ratio (AOR): 2.56; p = 0.048), alcohol consumption (AOR: 1.85; p = 0.026), and consulting healthcare professionals (AOR: 1.96; p = 0.026) were associated with contraceptive use. Also, 36 (13.3%) of the study participants had never been tested for STIs.

Conclusion

This study highlights that a relatively high prevalence of contraceptive usage and also higher socioeconomic status, alcohol consumption, and consulting healthcare professionals are associated with the use of contraceptives. Though the prevalence of contraceptive usage is high, there are some participants who did not consult healthcare professionals and have never been tested for STIs. These findings can help to plan better awareness programs and sexual health practices.

## Introduction

In the digital age, online dating apps play a vital role in social interactions, especially among young adults. The age group 18-35 has strong emotional and social needs, making these platforms essential for connection. Users seek companionship, like-minded individuals to explore their fantasies and sexual desires. Research indicates that dating apps contribute to self-discovery and relationship-building. The COVID-19 pandemic further increased app usage, as lockdowns heightened the need for virtual interactions. This shift emphasized the growing influence of digital platforms in shaping modern relationships, fostering emotional connections, and providing a space for personal exploration [1]. Few studies have defined a dating app as a smartphone-based digital platform that enables users to create profiles with images and browse others' profiles. These apps allow users to search for potential matches based on shared interests, preferences, or location and communicate with them directly [2,3].

In India, the use of dating apps has increased significantly among unmarried young adults. Chennai, being one of the country’s major urban hubs, reflects this trend with a growing number of adults actively engaging in online dating. These apps have become a popular way for young people to connect [4], helping them overcome feelings of loneliness and a lack of social interaction. The widespread use of dating apps provides an outlet for forming new relationships, making social connections, and fulfilling emotional needs, especially in today's fast-paced, digitally driven world. 

The leading dating apps across India are Tinder (66%), Bumble (41%), Hinge (27%), OK Cupid (26%), and others like Happn and Grindr, which are all less than 25% [5]. A statistic states that nearly 60 million monthly users use dating apps like Tinder. In which nearly 60% of Tinder users are under the age group of 35 years, and the majority of the users are male [6]. 

Research has shown that dating app usage is linked to various sociodemographic and personality traits. Traditionally, men were believed to use these apps more frequently. While men engage more actively, women tend to use them more selectively and effectively, leading to more successful matches [7]. Most studies focus on college students aged 18-24, with research indicating that around 40% of students use Tinder. However, some studies suggest that the average user's age may be closer to 31. Therefore, a direct relationship between college students’ age and dating app usage is expected. Its frequency is not only increased among men and women but has also influenced other groups like the lesbian, gay, and LGBTQ community [8-12].

The problematic use of online dating apps includes mental health disorders like mood modification, tolerance, conflict, withdrawal, lower self-esteem, low levels of bodily satisfaction, anxiety, depression, addiction, and negative behaviors and has also led to more communicable diseases like sexually transmitted diseases due to sexual act and fantasies.

Nowadays, the majority of gay, bisexual, and other men who have sex with men (MSM) utilize dating apps to find romantic and sexual partners. Dating apps make it simple to locate new partners and are often accessible for free, unlike in bars or clubs. Because they facilitate the rapid discovery of new partners, they can also broaden sexual networks. They can accomplish this in locations where dating apps might be the only way to locate partners, as well as in larger cities that already have a number of ways to meet them. Furthermore, a number of studies have discovered that men, women, and MSM who connect with partners online are more likely to engage in condom-free sex, have more male partners, engage in higher-risk sex behaviors, and have higher rates of STDs [13]. It is evident that COVID-19-related disruptions affected a number of MSM and other groups' behaviors, including altering their dating app usage patterns [14]. Contraception refers to methods that are used to prevent pregnancy, including devices, medications, procedures, or behaviors. Barriers for effective contraceptive use include limited access, restricted method choices, fear of side effects, cultural or religious beliefs, poor healthcare services, and gender-based challenges [15]. The primary objective of this study is to estimate the prevalence and to determine the various factors affecting Chennai's online dating app users' use of contraceptives.

## Materials and methods

Study design

This is a community-based cross-sectional study.

Study duration

This study was conducted for six months from October 2024 to March 2025 in Chennai, Tamil Nadu.

Sample size

The sample size was determined based on a cross-sectional study done by Dmello et al. [16], which reported that 34.7% of the population were aware about the usage of condoms and other preventive measures. Using this prevalence, with an absolute precision of 6%, the estimated sample size for the study was calculated to be 270.

Sampling technique

The snowball sampling technique was used for the selection of participants.

Inclusion criteria

This study included individuals aged 18 and above who were using online dating apps for the past six months, residing in Chennai.

Data collection

A pretested, semi-structured questionnaire was developed based on a review of the literature. A pilot study was conducted among 25 participants, and with the help of expert opinions in public health, necessary modifications were made to the questionnaire. The participants from the pilot study were excluded from the final analysis. The reliability of the questionnaire was assessed, yielding a Cronbach’s alpha of 0.7. The final validated questionnaire was used to collect data on the usage of contraceptives among online dating app users.

Data analysis

Data were collected, entered, and analyzed using MS Excel (Microsoft Corporation, Redmond, Washington, United States) and IBM SPSS Statistics for Windows, Version 21 (Released 2012; IBM Corp., Armonk, New York, United States).

Statistical analysis

Descriptive statistics were presented using numbers and percentages. The association between sociodemographic variables, awareness, and various factors associated with the usage of contraceptives was assessed using analytical statistics, and the odds ratio was used to establish the statistical significance at a 95% confidence interval. Binary logistic regression analysis was performed to identify independent predictors of contraceptive usage.

Ethical approval

Ethical clearance was obtained from the Institutional Human Ethical Committee (002/SBMCH/IHEC/2024/2329) of a tertiary care hospital.

## Results

The study surveyed 270 participants, and most of the participants were in the age group of 25-34 years (163, 60.3%), with the majority of participants being male (160, 59.3%). Most of them resided in urban areas (215, 79.6%). Nearly half of them were undergraduates and graduates (132, 48.9%), and most of the participants were living with their father, mother, and sister/ brother. Most of the participants were students (108, 40%) and employed individuals (104, 38.5%). Among the study participants, many were single (88, 32.6%) or in a relationship (79, 29.3%). Most belonged to upper or upper-middle socioeconomic classes (249, 92.3%), and over half lived in two-room houses (140, 51.9%), as shown in Table 1. 

**Table 1:** Sociodemographic details of the study participants (n = 270)

Variable	Category	Frequency (n = 270)	Percentage (%)
Age in years	18-24	99	36.6
	25-34	163	60.3
	35-44	8	2.96
Gender	Male	160	59.3
	Female	110	40.7
Place of living	Urban	215	79.6
	Rural	55	20.4
Living arrangements	Living with father, mother, and sister/brother	97	35.9
	Living with father, mother, sister/brother, with their spouse	65	24.1
	Living with father, mother, grandparents, and siblings	21	7.8
	Living with parents, grandparents, married brother or sister in the same house	19	7
	Living alone/living only with dad/ living with mom/others	68	25.2
Educational qualification	High school	27	10
	Higher secondary	24	8.9
	Undergraduate/graduate	132	48.9
	Post graduate	87	32.2
Employment status	Student	108	40
	Unemployed	32	11.9
	Employed	104	38.5
	Part-time jobs/business	26	9.6
Religion	Hindu	176	65.2
	Muslim	51	18.9
	Christian	43	15.9
Marital status	Single/not dating	88	32.6
	In a relationship	79	29.3
	Married	35	13
	Separated/divorced	12	4.4
	Situationship/benching/living together/casual relationship	56	20.7
Socioeconomic classification [17]	Class 1 (upper class)	123	45.6
	Class 2 (upper middle class)	126	46.7
	Class 3 (middle class)	10	3.7
	Class 4 (lower middle class)/class 5 (lower class)	11	4.1
Number of rooms in the place of living	One	54	20
	Two	140	51.9
	Three	76	28.1

**Figure 1:**
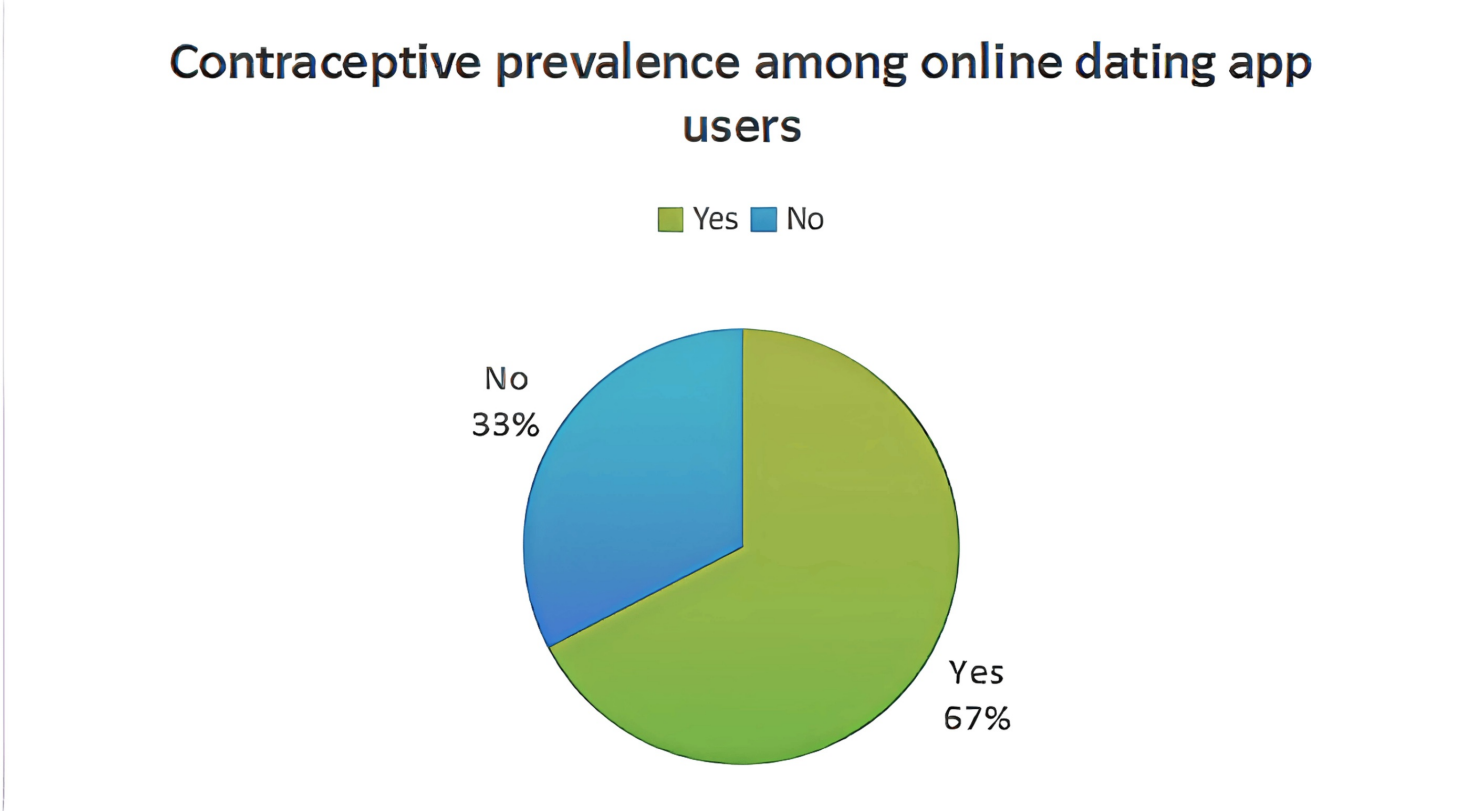
Contraceptive prevalence among online dating app users (n = 270)

Figure 1 shows the contraceptive prevalence among online dating app users. The majority of the study participants reported using contraceptives (182, 67%), while the rest of the participants did not use contraceptives (88, 33%).

Table 2 shows the assessment of 270 individuals regarding their level of contraceptive awareness and their usage behavior. The majority of participants knew that contraception promoted safe sexual practices (117, 43%), while 96 (36%) knew it prevented pregnancy. Condoms were the most common method known (130, 48%), followed by combinations with emergency contraception (76, 28%). Friends and family (94, 35%) and internet platforms/media (88, 33%) were the most common information sources. Only a small percentage (25, 9%) stated healthcare providers as an information source. About 120 (44.4%) use online dating apps on a weekly basis. The majority of the study participants (182, 67.4%) have met <5 sexual partners through online dating apps. 

**Table 2:** Awareness and usage behavior of contraceptives by the study participants (N = 270)

Variables	Category	Frequency (N = 270)	Percentage (%)
Perception toward contraception	It is a preventive measure for pregnancy	96	36
	It involves methods that promote safe sexual practices	117	43
	It is a health strategy for population control	38	14
	It has both medical and natural approaches	12	4
	I am not familiar with the concept of contraception	7	3
Contraceptive methods known	Condoms	130	48
	Condoms and emergency contraception	76	28
	Condoms and natural methods	27	10
	Intrauterine devices, oral contraceptive pills, implants/injections	37	14
Source of information about contraceptives	Friends/family	94	35
	School/college	63	23
	Online platforms, media/advertisements	88	33
	Healthcare providers	25	9
Frequency of using dating apps	Daily	68	25.2
	Weekly	120	44.4
	Monthly	69	25.6
	Rarely	13	4.8
No. of sexual partners met through dating apps	<5	182	67.4
	>5	88	32.6

**Figure 2:**
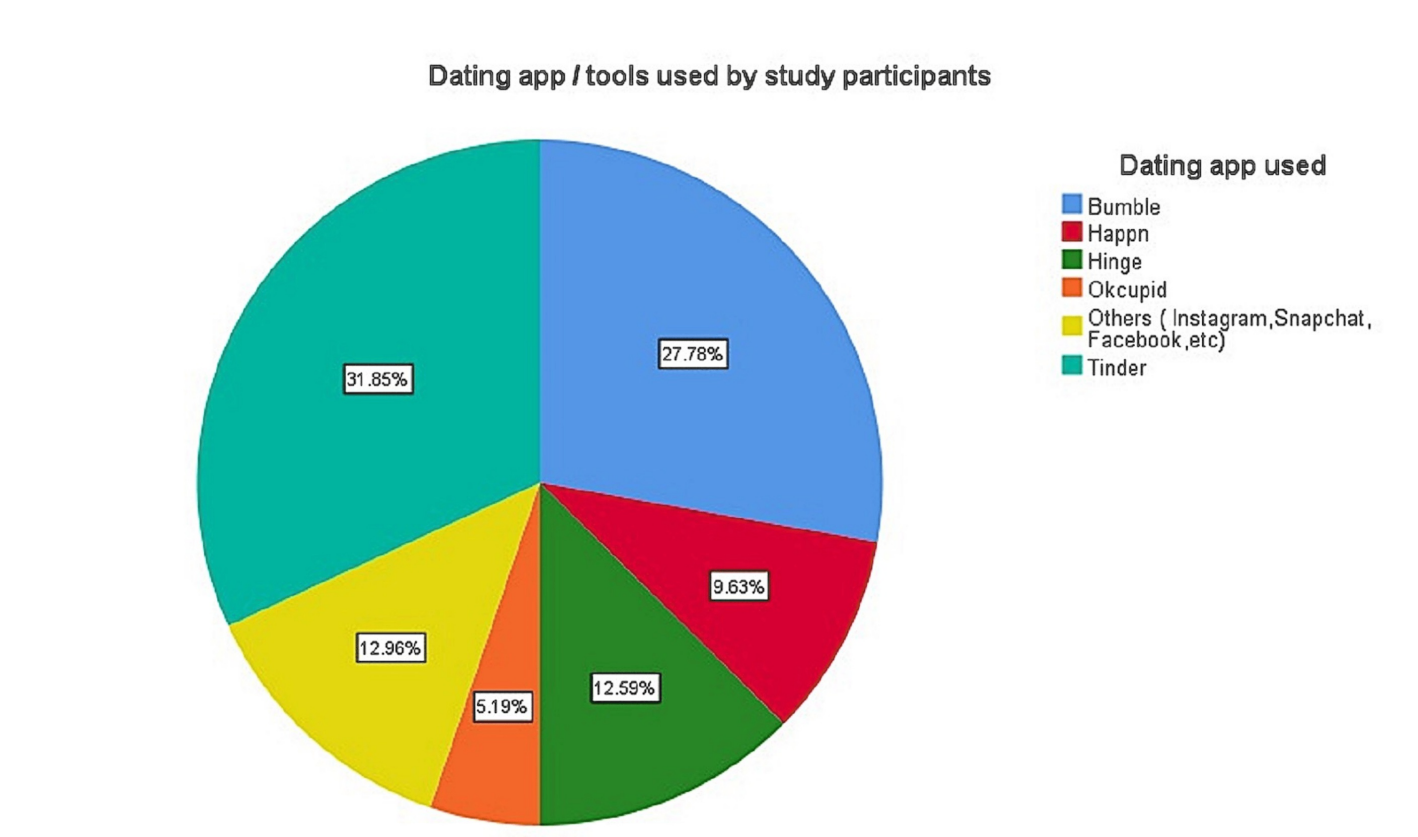
Various dating apps/tools used by study participants (n = 270)

Figure 2 illustrates the various dating apps used by the study participants. Tinder was the most widely used app (31.85%), followed by Bumble (27.78%). The other dating apps, like Instagram, Snapchat, Facebook, Hinge, and Happn, were all used relatively less.

Table 3 shows a binary logistic regression analysis of various factors associated with contraceptive use among online dating app users. Younger age (≤26 years) was found to be significant; however, it was not found statistically significant after adjusting for the odds ratio. The upper/upper-middle socioeconomic class (AOR: 2.56; p = 0.048), and alcohol consumption (AOR: 1.85; p = 0.026) were associated risk factors with contraceptive use. Participants who are not consulting healthcare professionals are at increased risk of not using contraceptives (AOR: 1.96; p = 0.026). 

**Table 3:** Binary logistic regression of various factors associated with contraceptive usage among online dating app users Ref: reference category; * represents p-value of <0.05, which is considered to be statistically significant; unadjusted OR with 95% confidence interval (CI). The significant factors that satisfied p < 0.05 were considered for binary logistic regression analysis, which is represented with adjusted odds ratio (AOR) with 95% CI

Variables	Usage of contraceptives among dating app users		Total (n = 270),(%)	Unadjusted OR (95% CI)	p-value of unadjusted OR	Adjusted OR (95% CI)	p-value of adjusted OR
	Yes (n,%)	No (n,%)					
Age							
Upto 26 years	105 (38.8)	40 (14.8)	145 (54)	1.636 (0.980-2.732)	0.04	-	-
Above 26 years	77 (28.5)	48 (17.7)	125 (46)	Ref	-	-	-
Socioeconomic classification [17]							
Upper class/upper middle class	172 (63.7)	77 (28.5)	249 (92)	2.457 (1.001-6.029)	0.04	2.560 (1.008-6.502)	0.048*
Middle class/lower middle class/lower class	10 (3.7)	11 (4)	21 (8)	Ref	-	Ref	-
Smoking							
Yes	115 (42.5)	49 (18)	164 (61)	1.366 (0.814-2.292)	0.237	-	-
No	67 (24.8)	39 (14)	106 (39)	Ref	-	-	-
Alcohol consumption							
Yes	89 (32.9)	31 (11.4)	120 (44)	1.760 (1.041-2.975)	0.034	1.852 (1.078-3.181)	0.026*
No	93 (34.4)	57 (21)	150 (56)	Ref	-	Ref	-
Contraceptives are effective in preventing pregnancies							
No	55 (20.3)	0	55 (20)	1.693 (1.515-1.892)	0.000	-	-
Yes	127 (47)	88 (32.5)	215 (80)	Ref	-	-	-
Specified radius							
1-10 kms	80 (29.6)	27 (10)	107 (40)	1.772 (1.033-3.039)	0.037	-	-
11-25 kms	102(37.7)	61(22.5)	163 (60)	Ref	-	-	-
Consulting healthcare professionals							
No	74 (27.4)	22 (8.1)	96 (36)	2.056 (1.167-3.620)	0.012	1.962 (1.084-3.550)	0.026*
Yes	108 (40)	66 (24.4)	174 (64)	Ref	-	Ref	-
Reasons for using dating apps							
Boredom, casual dating, to find friends & long-term relationship	119 (44)	40 (14.8)	159 (59)	2.267 (1.349-3.809)	0.002	-	-
Casual sex & self-confidence boost	63 (23.3)	48 (17.7)	111 (41)	Ref	-	-	-
Place of living							
Urban	145 (53.7)	71 (26.2)	216 (80)	0.938 (0.494-1.781)	0.846	-	-
Rural	37 (13.7)	17 (6.2)	54 (20)	Ref	-	-	-
Privacy in the place of living							
Present	141 (52.2)	67 (24.8)	208 (77)	1.078 (0.591-1.966)	0.807	-	-
Not present	41 (15)	21 (7.7)	62 (23)	Ref	-	-	-

Table 4 shows that most of the study participants had consulted healthcare professionals regarding contraceptive options (174, 64.4%). While 121 (44.8%) reported occasional sexually transmitted infection (STI) testing, only 43 (15.9%) did so regularly. However, 36 (13.3%) had never been tested for STIs. About 215 (79.6%) participants felt that dating apps positively influence contraceptive use. 

**Table 4:** Health-seeking behavior of online dating app users (n = 270)

Variables	Category	Frequency (n = 270)	Percentage (%)
Frequency of getting tested for sexually transmitted infections	Never	36	13.3
	Rarely	70	25.9
	Occasionally (once a year)	121	44.8
	Regularly (every 3-6 months)	43	15.9
Consultation of healthcare professionals regarding contraceptives options	No	96	35.6
	Yes	174	64.4
Online dating apps contributes positively to the usage of contraceptives	No	55	20.4
	Yes	215	79.6

## Discussion

With the increasing popularity of online dating apps, the fantasy of romantic and sexual relationships has shifted dramatically in recent years. These platforms have grown in popularity among people looking for both casual and long-term relationships, especially in urban areas like Chennai, Tamil Nadu.

In our study, males (160, 59.3%), single (88, 32.6%), and those in the age group 25-34 (163, 60.3%) were found to be using online dating apps more. Similarly, in a study done by Blanc, it was found that the highest number of online dating app users are males [18]. Another study done in Spain [8] shows that older youths and being single/open-mindedness contributed to higher usage of online dating apps. The similarities in this study, when compared to other studies, are because males usually tend to initiate contact through online dating apps more than females. Also, single or open-minded youths of the age group 25-34 are more inclined to online dating because they are in constant need of relationships and companionship.

In our study, the prevalence of contraceptive use among online dating app users was found to be 182 (67%). Similarly, a study by Stewart et al. has shown that 82% of men have used contraceptives while using online dating apps [19]. Another study done in China shows that the contraceptive use of women using dating apps was found to be 78.9% [20]. Users of dating apps are most probably from urban areas, are well-educated, and have digital knowledge, which aids them in accessing better sexual information. They know all the risks associated, like STIs and unplanned pregnancies due to unprotected sex and multiple sexual encounters, thus leading to better usage of contraceptives. In contrast, a study done by Reeves et al. shows that dating app users engage in more condomless sexual activity [21]. Also, another study done in the United States of America shows that there is less prevalence of contraceptive use among online dating app users [22]. Factors like risky behavior, social stigma, barriers to accessing contraceptives, and substance use may impair the usage of contraceptives. Not all people are health-conscious, and those seeking long-term relationships find it difficult to use contraceptives as they prioritize spontaneous and recreational encounters.

In our study, the usage behavior of dating app users was high on a weekly basis (120, 44.4%), the number of partners met through dating apps is <5 sexual partners (182, 67.4%), and the specified radius is 11-25 km (160, 59.3%). Similarly, a study conducted in the United States of America shows that the average number of sexual partners is 5 [23]. Users practiced constant use of online dating apps while being cautious of the high risk associated with sexual practices. Many users don’t want their partners to be nearby, like within 10 km, and want an expanded potential match over a greater radius. This may be due to broader safety concerns and trust, which are universal considerations. Conversely, a study done by Goedel et al. stated that daily usage is higher among online dating app users [24]. In contrast, a study done by Algarin et al. shows that online dating app users prefer partner-seeking within a one-mile radius [25]. The population with higher engagement in online dating apps prefers their sexual partners to be found in a close proximity radius that is within a one-mile radius, and they use dating apps on a daily basis. Certain geosocial dating apps like Grindr make use of nearby proximity for convenience in densely populated areas. 

In our study the people of upper/upper-middle socioeconomic class were considered a risk factor for contraceptive use. In a meta-analysis done by Islam et al. showed that those belonging to upper class of the socioeconomic scale had higher chances of using contraceptives [26]. Even though the upper socioeconomic class participants have better education, the lower usage of contraceptives may be due to partner preference or side effects.

In our study, out of the study participants, 121 (44.8%) reported occasional STI testing, and only 43 (15.9%) did so regularly. However, 36 (13.3%) had never been tested for STIs. Similarly, a study conducted by Parker et al. stated that 35.5% of the study participants never got tested for STIs [27]. Another study done by Frankis et al. shows that only one-third of the participants got tested regularly for STIs [28]. Social stigma, discrimination, lack of access, and cultural norms all hinder the individual's ability to get tested for STIs and also the usage of contraceptives.

Limitations

As it is a cross-sectional study, which relies on self-reported data, it brings about social desirability and recall biases, which may affect accuracy. A causal relationship cannot be established due to the study’s design. The sample may not represent all the online dating app users in Chennai due to selection bias.

Recommendation

Strengthening targeted contraceptive awareness initiatives among online dating app users, with a particular focus on those from lower socioeconomic backgrounds, is recommended. Initiatives for health education ought to emphasize the importance of consulting with healthcare professionals in order to make educated decisions about contraception. Incorporating information about sexual and reproductive health in online dating apps could be a strategic intervention.

## Conclusions

This study highlights a relatively high prevalence of contraceptive usage among individuals in Chennai, Tamil Nadu, who use online dating apps. Higher socioeconomic status, alcohol consumption, and consulting healthcare professionals were all substantially associated with the use of contraceptives. Though the prevalence of contraceptive usage among dating app users is high, a portion of participants did not consult healthcare professionals, and a few participants never or rarely get tested for sexually transmitted diseases. These findings highlight the necessity of focused awareness programs and easily available sexual health services for the diverse online dating app-using populations.

## Appendices

**Table 5:** Questionnaire IUDs: intrauterine devices

S. no.	Response
1.	Age:
	18-24 years
	25-34 years
	35-44 years
2.	Gender:
	Male
	Female
3.	Place of living:
	Rural
	Urban
4.	Number of family members
5.	Total income of the family
6.	Living arrangements:
	Living with father, mother, and sister/brother
	Living with father, mother, sister/brother, with their spouse
	Living with father, mother, grandparents, and siblings
	Living with parents, grandparents, married brother or sister in the same house
	Living alone/only with dad/with mom/others
7.	Educational qualification:
	High school
	Higher secondary
	Undergraduate/graduate
	Postgraduate
8.	Employment status:
	Employed
	Unemployed
	Student
	Part-time jobs/business
9.	Religion:
	Hindu
	Muslim
	Christian
10.	Marital status:
	Single/not dating
	In a relationship
	Married
	Separated/divorced
	Situationship/benching/living together/casual relationship
11.	Alcohol consumption:
	Yes
	No
12.	Smoking habit:
	Yes
	No
13.	Number of rooms in your place of living:
	One
	Two
	Three
14.	Privacy in your place of living:
	Present
	Not present
15.	Frequency of using online dating apps:
	Daily
	Weekly
	Monthly
	Rarely
16.	Understanding of contraception:
	Preventive measures for pregnancy
	Promotes safe sexual practices
	Health strategy for population control
	Medical and natural approaches
	Not familiar with the concept
17.	Awareness of contraceptive methods:
	Condoms
	Condoms & emergency contraception
	Condoms & natural methods
	IUDs, oral pills, implants/injections
18.	Source of information about contraceptives:
	Friends/family
	School/college
	Online platforms/media/ads
	Healthcare providers
19.	Do you believe contraceptives are effective in preventing unwanted pregnancies?
	Yes
	No
20.	Reasons for using dating apps:
	Boredom, casual dating, to find friends & long-term relationship
	Casual sex & self-confidence boost
21.	Dating apps/tools used:
	Tinder
	Bumble
	Happn
	Hinge
	OkCupid
	Others (Instagram, Snapchat, Facebook, etc)
22.	Frequency of dating app usage:
	Daily
	Weekly
	Monthly
	Rarely
23.	Number of sexual partners met via app:
	<5
	>5
24.	Specified radius on dating apps:
	1–10 km
	11–25 km
25.	Have you ever used contraceptives?
	Yes
	No
26.	How often do you get tested for STIs?
	Regularly (every 3-6 months)
	Occasionally (once a year)
	Rarely
	Never
27.	Consult healthcare professionals about contraception?
	Yes
	No
28.	Do dating apps increase the importance of contraceptive use/infection prevention?
	Yes
	No
